# Editorial: Breeding innovations in underutilized temperate fruit trees, volume II

**DOI:** 10.3389/fpls.2022.1111454

**Published:** 2022-12-13

**Authors:** Giuseppe Ferrara, Agata Gadaleta, Malli Aradhya, Jose Inaki Hormaza, Maria Luisa Badenes

**Affiliations:** ^1^ Department of Plant, Soil and Food Science, University of Bari ‘Aldo Moro’, Bari, Italy; ^2^ Agricultural Research Service, United States Department of Agriculture, Washington D.C., United States; ^3^ Department of Subtropical Fruit Crops. Instituto de Hortofruticultura Subtropical y Mediterranea La Mayora (IHSM-CSIC-UMA), Algarrobo, Malaga, Spain; ^4^ Departamento de Citricultura y Producción Vegetal. Instituto Valenciano de Investigaciones Agrarias, Moncada, Spain

**Keywords:** polyploidy, *Citrus*, *Rubus*, pomegranate, minor species

This Research Topic is dedicated to the memory of our coauthor Dr. Maria Luisa Badenes, a dear colleague and friend who prematurely passed away in September 2^nd^, 2022. Marisa was a brilliant scientist that during her scientific career has made highly relevant contributions in genetics and breeding of diverse fruit tree crops, such as apricot, peach, loquat, kiwi or persimmon. But, even more important, Marisa has been a generous colleague and an excellent person whose work will continue in the hands of the numerous scientists that have been mentored under her guidance or have collaborated with her. We all in the fruit breeding community will surely miss her.

The recent growing interest in minor species (i.e., in the genus *Punica*, *Rubus*, *Ficus*, *Citrus*, etc.) to diversify fruit production, even including health aspects, has attracted research towards breeding and genetics of various ‘forgotten’, ‘underutilized’ or ‘neglected’ fruit tree crop species (Giancaspro et al., 2017; Marcotuli et al., 2019; Marcotuli et al., 2020). Cultivation of such species in different areas of the world, through the development of new cultivars and improved traits, could be an important healthy and cost-effective alternative contributing to nutritional security. These species can be managed by using sustainable cultural practices, i.e., from irrigation with alternative waters (Bedbabis et al., 2014) to either the use of organic compounds to face water scarcity (Boselli et al., 2019) or even reduce chemical toxicity (Ferrara et al., 2000, 2004, 2006). Minor temperate fruit tree species have suffered of little attention from the scientific community, consequently receiving little improvement *via* conventional breeding, and detailed genomic information on important traits is still lacking. This lack of data, together with a general poor genetic knowledge of these species, has often limited a wider cultivation and returns for sustainable fruit production.

For these reasons, and in the eyes of a growing awareness of scientists, farmers and consumers, the present Research Topic ‘*Breeding innovations in underutilized fruit trees, volume II*’ has continued the collection of studies (started with volume I) on underutilized fruit tree species with regards of genetic aspects. In this context, Sabooni and Gharaghani studied polyploidy as an important phenomenon in the evolution of fruit crops. Polyploidy can be used in fruit breeding programs to develop varieties with higher yields and better fruit quality, as well as with better adaptation to adverse environmental conditions. In their study, the polyploidy of 3 wild blackberry species was investigated through different degrees of induced polyploidy. Data showed that polyploid plants performed significantly better than diploid ones, in particular for traits such as flower number per spike, drupe number per fruit and berry weight. On the other side, microscopic observations revealed a lower number of viable pollen grains in the polyploidy plants with respect to diploid ones. Moreover, polyploidy affected some biochemical traits (i.e., IAA/jasmonic acid ratio, TSS), number of pistils, leaf green index, TSS in the leaves, and glucose content in the floral nectar. Overall, induced polyploidy allowed *Rubus* to develop advantageous traits that can benefit future breeding programs and expand reproductive research for such species.

The topic of resistance was carried out by Alves et al. in order to find a full genetic resistance to Huanglongbing (HLB), the most destructive citrus disease worldwide, caused by phloem-limited *Candidatus liberibacter* species, mainly known as *Ca. L. asiaticus* (Las), which is transmitted by the insect *Diaphorina citri*. Some Oceanian citrus genotypes were evaluated as possible tolerant rootstocks for sweet oranges. Results showed that, after insect feeding on plants from the Oceanian citrus genotypes, quantitative real-time polymerase chain reaction (qPCR) data were negative. Moreover, their budwood material was unable to infect sweet orange plants through grafting. Furthermore, leaves from sweet oranges grafted on susceptible rootstocks resulted infected after insect feeding. From the evolutionary aspect, genomic and morphological analysis of the Oceanian genotypes confirmed that *E. glauca* and *M. warburgiana* are pure species while the *M. australis* accession is an *M. australis* × *M. inodora* hybrid and *M. papuana* is probably a *M. papuana* × *M. warburgiana* hybrid. An *E. glauca* × *C. sinensis* hybrid was found to originate from a cross between *E. glauca* and either mandarin or tangor. *Eremocitrus* × *Microcitrus* hybrid is a complex admixture of *M. australasica*, *M. australis*, and *E. glauca* while the last hybrid is an *M. australasica* × *M. australis* admixture.

Pomegranate (*Punica granatum* L.) was investigated by Patil et al. for the identification and utilization of gene-based marker systems. The authors identified and designed a total of 8,812 potential intron polymorphism (PIP) markers specific to 3,445 (13.40%) gene models that span 8 chromosomes in a Tunisia variety. The ePCR validation of all these PIP markers on the Tunisia genome revealed single-locus amplification for 1,233 (14%) markers corresponding to 958 (27.80%) genes. Through ePCR, 1,233 PIP markers were assayed on multiple genomes, which resulted in the identification of 886 polymorphic markers with an average PIC value of 0.62. Authors demonstrated the potential utility of the developed markers by analyzing the genetic diversity of 31 pomegranate genotypes using 24 PIP markers. This study reports for the first time a large-scale development of gene-based and chromosome-specific PIP markers, which could serve as a rich marker resource for genetic variation studies, functional gene discovery, and genomics-assisted breeding of pomegranate.

Pomegranate was also studied by Sowjanya et al. with regards of *de novo* whole-genome sequencing of the main Indian cultivar ‘Bhagawa’. The research led to a final reference-quality genome assembly for ‘Bhagawa’ of 346.08 Mb in 342 scaffolds and an average N50 of 16.12 Mb and N90 of 1088.62 Kb. This assembly covered more than 98% of the estimated pomegranate genome size, 352.54 Mb. The LTR Assembly Index (LAI) value of 10 and 93.68% Benchmarking Universal Single-Copy Orthologs (BUSCO) completeness score over the 1,440 ortholog genes of the completed pomegranate genome indicates the good quality of the assembled pomegranate genome. Authors performed whole-genome phylogenetic analysis using Computational Analysis of Gene Family Evolution (CAFE) and found that *Eucalyptus grandis* and pomegranate diverged ≈64 (60–70) million years ago. Moreover, either 1,573 protein-coding resistance genes classified into 32 domains or 314 copies of miRNA belonging to 26 different families were identified in the ‘Bhagawa’ genome. The reference-quality genome assembly of ‘Bhagawa’ is certainly a significant genomic resource to accelerate pomegranate improvement.

## Author contributions

All authors listed have made a substantial, direct, and intellectual contribution to the work and approved it for publication.
